# Authentic CRAC channel activity requires STIM1 and the conserved portion of the Orai N terminus

**DOI:** 10.1074/jbc.M117.812206

**Published:** 2017-12-13

**Authors:** Isabella Derler, Carmen Butorac, Adéla Krizova, Michael Stadlbauer, Martin Muik, Marc Fahrner, Irene Frischauf, Christoph Romanin

**Affiliations:** From the Institute of Biophysics, Johannes Kepler University of Linz, Gruberstrasse 40, 4020 Linz, Austria

**Keywords:** calcium channel, calcium release-activated calcium channel protein 1 (ORAI1), electrophysiology, signal transduction, stromal interaction molecule 1 (STIM1), calcium-dependent inactivation, sodium permeation

## Abstract

Calcium (Ca^2+^) is an essential second messenger required for diverse signaling processes in immune cells. Ca^2+^ release-activated Ca^2+^ (CRAC) channels represent one main Ca^2+^ entry pathway into the cell. They are fully reconstituted via two proteins, the stromal interaction molecule 1 (STIM1), a Ca^2+^ sensor in the endoplasmic reticulum, and the Ca^2+^ ion channel Orai in the plasma membrane. After Ca^2+^ store depletion, STIM1 and Orai couple to each other, allowing Ca^2+^ influx. CRAC-/STIM1-mediated Orai channel currents display characteristic hallmarks such as high Ca^2+^ selectivity, an increase in current density when switching from a Ca^2+^-containing solution to a divalent-free Na^+^ one, and fast Ca^2+^-dependent inactivation. Here, we discovered several constitutively active Orai1 and Orai3 mutants, containing substitutions in the TM3 and/or TM4 regions, all of which displayed a loss of the typical CRAC channel hallmarks. Restoring authentic CRAC channel activity required both the presence of STIM1 and the conserved Orai N-terminal portion. Similarly, these structural requisites were found in store-operated Orai channels. Key molecular determinants within the Orai N terminus that together with STIM1 maintained the typical CRAC channel hallmarks were distinct from those that controlled store-dependent Orai activation. In conclusion, the conserved portion of the Orai N terminus is essential for STIM1, as it fine-tunes the open Orai channel gating, thereby establishing authentic CRAC channel activity.

## Introduction

Calcium (Ca^2+^) represents an important second messenger that is indispensable for various signaling processes in immune and other types of cells (1–3). The Ca^2+^ release-activated Ca^2+^ (CRAC)^3^ channel, which is activated following intracellular Ca^2+^ store depletion, represents one main Ca^2+^ entry pathway (4, 5).

CRAC channels are fully reconstituted via two proteins, the stromal interaction molecule 1 (STIM1) and Orai (5–12). STIM1 represents an endoplasmic reticulum (ER)-located Ca^2+^sensing protein (6, 7, 13), whereas Orai forms the Ca^2+^-selective ion channel in the plasma membrane (5–8, 14–17). Upon depletion of Ca^2+^ from the ER, STIM1 proteins oligomerize, move into discrete puncta at the plasma membrane (PM)-ER junctions, and couple to and activate Orai channels (16, 18–21). Subsequently, Ca^2+^ permeates the Orai channel to enter the cell (22, 23).

The Orai protein family includes three members, Orai1–3. They are all composed of cytosolic N- and C-terminal strands and four transmembrane domains (TM) connected via intracellular (TM2-TM3) or extracellular (TM1-TM2 and TM3-TM4) loops (17, 24, 25). Both N and C termini are required for STIM1-dependent Orai channel activity (20, 22, 26–30). The Orai C terminus forms the main binding site for STIM1 (20), whereas direct STIM1 binding to the N terminus is currently controversial (31).

Based on the crystal structure of *Drosophila* Orai, Orai Ca^2+^ ion channels are assumed to form hexameric complexes (32). STIM1-induced Orai channel pore opening involves a rotation of the hydrophobic region in TM1. However, it has so far remained unclear how this conformational change takes place. Mutagenesis studies have revealed that certain amino acids, like Gly^98^, Phe^99^, Val^102^, and Val^107^ in TM1 (33–36), but also other TM residues, such as Leu^138^ (37), Trp^176^ (38), Thr^184^ (36), and Pro^245^ (28), or residues between TM4 and the C terminus (*i.e.* Leu^261^-Val^262^-His^264^-Lys^265^) (31) contribute to the maintenance of the closed state, as their point mutation leads to constitutively open channels. For this reason, it has been hypothesized that the open state is established upon global rearrangement of TM helices after STIM1 binding (28, 39, 40).

CRAC/Orai channel currents exhibit a strongly inwardly rectifying current/voltage relationship with a reversal potential higher than +50 mV (41, 42), which indicates one typical CRAC channel hallmark. The permeability for Ca^2+^ is 1000 times larger than for Na^+^ (43). Orai channels conduct small monovalent ions, such as Na^+^, Li^+^, or K^+^, as long as the monovalent solution lacks divalent ions. Monovalent Orai currents are inhibited by Ca^2+^ concentrations in the micromolar range (43–48). Upon the switch from a Ca^2+^-containing to a divalent-free (DVF) Na^+^-containing solution, CRAC/Orai currents have been reported to increase 5-fold or even more (42, 46, 49–51), displaying another prominent CRAC channel hallmark. Orai channels are almost impermeable for the monovalent ion Cs^+^ because of Orai's very narrow pore diameter (46, 52). Due to CRAC channels' very low single channel conductance in the range of 1 picosiemens, single-channel current measurements have not been feasible so far. Recently, however, single-channel open states were visualized via optical recordings employing Orai1 proteins fused to a genetically encoded calcium indicator (53).

Another typical hallmark of Orai/CRAC channels represents fast Ca^2+^-dependent inactivation (FCDI), which reduces Ca^2+^ entry and thus displays an important feedback mechanism to tightly control intracellular Ca^2+^ concentrations (43, 54). FCDI occurs in all three Orai channels within the first 100 ms of a voltage step and more often happens in Orai3 compared with Orai1 or Orai2 (42, 55, 56). In Orai1, FCDI is followed by a late reactivation phase over the next 2 s, in contrast to Orai2 and Orai3 channels, which subsequently show a slower inactivation phase (42, 55).

In our study, which focused on these three CRAC channel hallmarks, we discovered that several constitutively active Orai1 and Orai3 mutants displayed authentic CRAC channel activity, but only in the presence of STIM1 and the conserved portion of the Orai N terminus. The structural requirements for STIM1 within this conserved N terminus were identical in constitutively active as well store-operated Orai channels.

## Results

### Maintaining CRAC channel permeation characteristics of constitutively active Orai mutants requires STIM1

**Figure 1.**
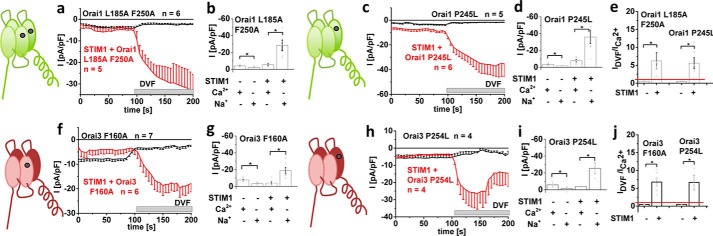
**CRAC channel permeation characteristics of constitutively active mutants are maintained in the presence of STIM1.** Shown are schemes illustrating Orai channels with the respective mutations tested: L185A/F250A and P245L in Orai1 and F160A and P254L in Orai3. *a*, *c*, *f*, and *h*, respective time courses of whole-cell inward currents at −74 mV of constitutively active mutants Orai1 L185A/F250A (*a*), Orai1 P245L (*c*), Orai3 F160A (*f*), and Orai3 P254L (*h*) in the absence compared with the presence of STIM1. At *t* = 0 s, inward currents in 10 mm extracellular Ca^2+^ solution activated upon passive store depletion via 20 mm EGTA are shown after reaching a steady-state level, and after 100 s, Na^+^-DVF solution was perfused. *b*, *d*, *g*, and *i*, block diagram exhibiting current density at 50 and 150 s of tested mutants (*a*, *c*, *f*, and *h*) with and without STIM1. *e* and *j*, block diagram exhibiting the ratio of *I*_DVF_ *versus I*_Ca_^2+^ of tested mutants with and without STIM1 (*a*, *c*, *f*, and *h*). *Error bars*, S.E.; *, *p* < 0.05.

**Figure 2.**
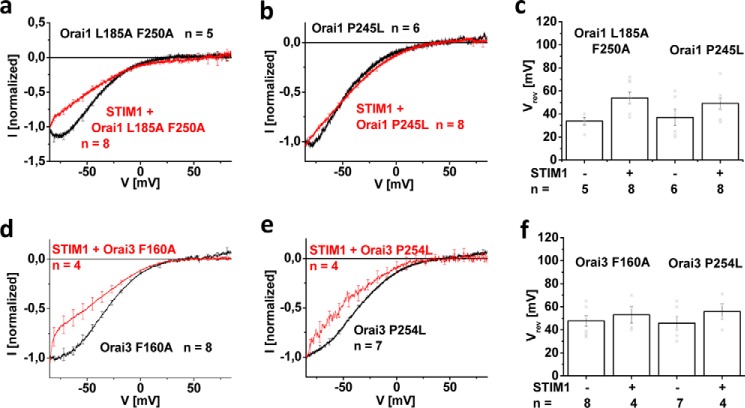
**Maintenance of typical CRAC channel *I*/V relationships of constitutively active Orai mutants requires STIM1.** *a*, *b*, *d*, and *e*, normalized *I*/V relationships of mutants tested in Fig. 1 in the absence compared with the presence of STIM1. *c* and *f*, reversal potentials of the respective mutants in *a*, *b*, *d*, and *e. Error bars*, S.E.; *, *p* < 0.05.

One of the major hallmarks of CRAC channel permeation is the increase in current density when switching from a Ca^2+^-containing to a DVF Na^+^-containing solution, as exemplified for wildtype Orai1 as well as wildtype Orai3, each co-expressed with STIM1 (Fig. S1). Here we focused on several constitutively active Orai1 and Orai3 channels, the activity of which become STIM1-independent by mutating one or two residues in TM3 and/or TM4. For Orai1, we specifically examined the Orai1 L185A (TM3)/F250A (TM4) double and the Orai1 P245L (TM4) single mutant, where the latter is associated with tubular myopathy (57). Both Orai1 mutants gave rise to robust, constitutive activity without STIM1 being co-expressed (Fig. 1), and they exhibited considerable yet somewhat reduced Ca^2+^ selectivity (see Fig. 2 and Fig. S2) in a 10 mm Ca^2+^-containing extracellular solution, when compared with the wildtype CRAC current. Strikingly, when testing for authentic CRAC channel permeation characteristics by switching from the Ca^2+^ to a DVF Na^+^-containing solution, a clear decrease in current density occurred with each constitutively active Orai1 channel mutant (Fig. 1 (*a–e*) and Fig. S2), in sharp contrast to what was seen in wildtype CRAC channels (compare with Fig. S1). Co-expression of these constitutively active Orai1 channel mutants with STIM1, however, dramatically altered their response to DVF Na^+^-containing solution, given that the expected ∼5-fold increase in current density (Fig. 1*e*) occurred in line with the hallmark of wildtype CRAC channels. Although we have only seldom detected the reported (43–45, 47) deactivation of STIM1/Orai-mediated Na^+^ currents, which is probably due to a slow solution exchange, these STIM1-dependent, maximum current increases of constitutively active Orai mutants in DVF solution have been seen in both N-terminally and C-terminally labeled mutants. It was exemplarily shown for Orai1 P245L (Fig. S3, *a–c*), thus excluding any side effect from the label. Constitutive Orai1 currents were not distorted by endogenous STIM1, as overexpression of constitutive Orai1 mutants in CRISP/Cas9 STIM1 knockout HEK cells revealed comparable behaviors whether in the absence or presence of STIM1, as exemplarily shown for Orai1 P245L (Fig. S4, *a–c*). Furthermore, disruption of potential STIM1 binding by the introduction of the Orai1 L273D C-terminal mutation (58) did not alter activity, as exemplified by the Orai1 P245L/L273D mutant expressed in HEK 293 cells (Fig. S4, *d–h*).

To assess the Orai isoform specificity of the observed dependence on permeation characteristics in the presence of STIM1, we further tested two constitutively active Orai3 channel mutants (*i.e.* Orai3 F160A and Orai3 P254L). The latter represented the analogue of Orai1 P245L, and both exhibited robust, constitutive activity in the absence of STIM1. As with the Orai1 mutants, switching from the Ca^2+^-containing to the DVF Na^+^-containing solution resulted in a decrease of current density in the absence of STIM1, whereas in the presence of STIM1, current densities were increased (Fig. 1, *f–j*), as expected for CRAC channel–like hallmarks.

Hence, authentic CRAC channel behavior of constitutively active Orai1 or Orai3 isoforms with respect to di-/monovalent permeation characteristics required their coupling to STIM1. Overall, the results indicate that the Orai channel open state elicited by specific point mutations in TM3 and/or TM4 retained CRAC channel-like permeation properties only in the presence of STIM1.

### STIM1 is required to maintain authentic CRAC channel current/voltage relationship

CRAC currents typically display a strongly inwardly rectifying current/voltage (*I*/V) relationship with a positive slope conductance at negative potentials and a reversal potential higher than +50 mV. Fig. 2 displays the *I*/V relationships of the constitutively active Orai1 and Orai3 mutants, introduced in Fig. 1, both in the absence and presence of STIM1. Voltage ramps over a duration of 1 s were applied at a holding potential of 0 mV, ranging from −90 to +90 mV, and for better comparison, the depicted *I*/V relationships were normalized to −1 at −85 mV. All Orai channel mutants exhibited strongly inwardly rectifying current/voltage relationships without STIM1 co-expressed; however, they revealed a U-shaped form at very negative potentials between −85 and −70 mV (Fig. 2). The reversal potentials of the Orai1 and Orai3 mutants were in the range of ∼+35 and ∼+48 mV, respectively, with the Orai1 mutants typically exhibiting lower reversal potentials than Orai3 (Fig. 2, *c* and *f*). In the presence of STIM1, the *I*/V relationships regained inward rectification similar to that of wildtype STIM1/Orai1 and STIM1/Orai3 currents together with a rightward shift of the reversal potentials toward ∼+50 mV comparable with authentic CRAC channel currents (Fig. 2). The U-shaped characteristic of the *I*/V relationship of constitutively active mutants in the absence of STIM1 was not visible in a faster voltage ramp over 200 ms, as exemplarily shown for Orai3 F160A (Fig. S5), pointing to a mechanism that evolved at the time scale of 1 s (see below). Nonetheless, the permeation characteristics of Orai3 F160A when switching from an extracellular to a DVF solution similarly exhibited a dependence on STIM1, with a current decrease and increase in the absence and presence of STIM1, respectively. This indicated that the persistence of this CRAC channel hallmark was not dependent on the duration of the applied voltage ramp (Fig. S5).

In summary, constitutively active TM3 and TM4 Orai1 as well as Orai3 point mutants displayed inwardly rectifying *I*/V relationships with an unusual, U-shaped characteristic, as revealed by voltage ramps of 1-s duration. Only the presence of STIM1 readjusted *I*/V relationships both in form and reversal potential toward those of the wildtype forms. Next, we checked whether this U-shaped form in current/voltage relationships might be related to a change of current inactivation profiles.

### Ca^2+^-dependent Orai inactivation necessitates STIM1

**Figure 3.**
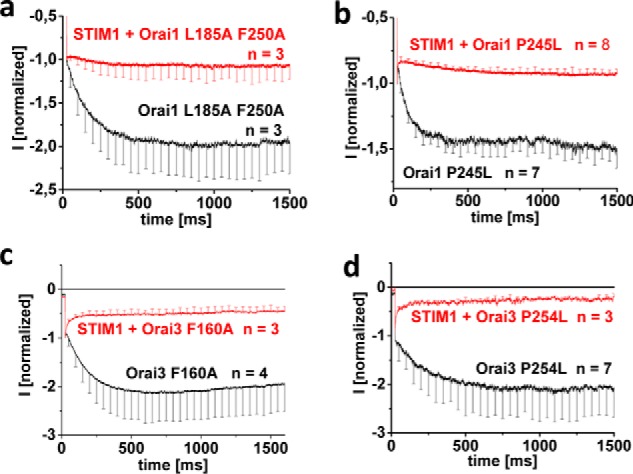
**CRAC channel-like inactivation of constitutively active Orai mutants requires STIM1.** *a–d*, inactivation characteristics of constitutively active mutants shown in Figs. 1 and 2 in the absence compared with the presence of STIM1. *Error bars*, S.E.

FCDI provides negative feedback to CRAC channels, a unique hallmark that is seen with both STIM1-activated Orai1 and Orai3 isoforms (Fig. S1, *f* and *l*). FCDI of the constitutively active Orai1/3 mutants was completely abolished in the absence of STIM1 (Fig. 3, *a–d*). Moreover, inactivation was not only blunted but was reversed into a robust potentiation, reaching a plateau between 500 and 1000 ms during a 1500-ms voltage step to −70 mV applied from a holding potential of 0 mV. The co-expression of STIM1 with these mutants fully restored FCDI to a level comparable with that of wildtype STIM1-mediated Orai1 and Orai3 currents (Fig. 3, *a–d*).

In aggregate, constitutively active Orai1 and Orai3 TM3/TM4 mutants required STIM1 coupling to retain the typical FCDI characteristics of authentic CRAC channels. Overall, STIM1 was indispensable in conveying the plethora of CRAC channel hallmarks onto the constitutively active Orai1/Orai3 channels, despite their propensity of STIM1-independent gating.

### STIM1 fails in maintaining CRAC channel hallmarks of constitutively active Orai channels with substantial N-terminal deletion

**Figure 4.**
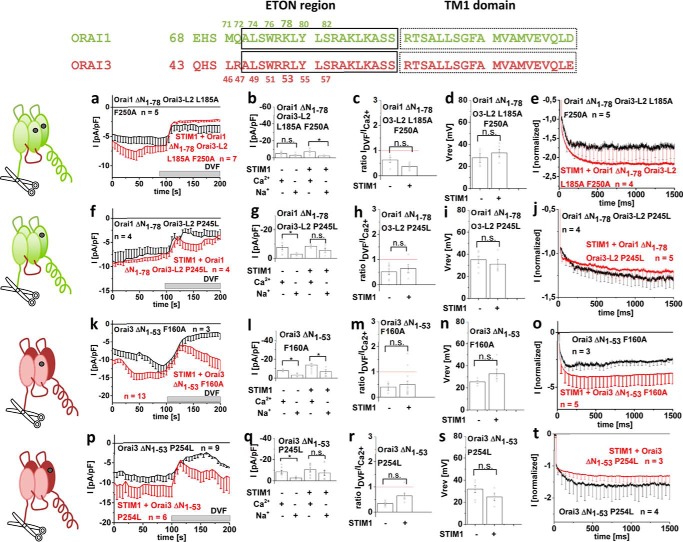
**STIM1 fails in maintaining CRAC channel hallmarks of constitutively active Orai channels with substantial N-terminal deletions.** Shown is a sequence alignment of Orai1 and Orai3 N terminus highlighting N-terminal residues at which the Orai N terminus was truncated. Shown are schemes illustrating the Orai channels with the respective mutations tested: Orai1 ΔN_1–78_ L185A/F250A Orai3-L2, Orai1 ΔN_1–78_ P245L Orai3-L2, Orai3 ΔN_1–53_ F160A, and Orai3 ΔN_1–53_ P254L. *a*, *f*, *k*, and *p*, respective time courses of whole-cell inward currents at −74 mV of constitutively active mutants: Orai1 ΔN_1–78_ L185A/F250A Orai3-L2 (*a*), Orai1 ΔN_1–78_ P245L Orai3-L2 (*f*), Orai3 ΔN_1–53_ F160A (*k*), and Orai3 ΔN_1–53_ P254L (*p*) in the absence compared with the presence of STIM1. At *t* = 0 s, inward currents in 10 mm extracellular Ca^2+^ solution activated upon passive store-depletion via 10 mm EGTA are shown after reaching a steady-state level, and after 100 s, Na^+^-DVF solution was perfused. *b*, *g*, *l*, and *q*, block diagram exhibiting current density at 50 and 150 s of tested mutants without and with STIM1 (*a*, *f*, *k*, and *p*). *c*, *h*, *m*, and *r*, block diagram exhibiting the ratio of *I*_DVF_ *versus I*_Ca_^2+^ of tested mutants without and with STIM1 (*a*, *c*, *k*, and *p*). *d*, *i*, *n*, and *s*, block diagram exhibiting the reversal potentials of the respective mutants in the absence compared with the presence of STIM1. *e*, *j*, *o*, and *t*, inactivation characteristics of constitutively active mutants shown in this figure in the absence compared with the presence of STIM1. *Error bars*, S.E.; *, *p* < 0.05; *n.s.*, not significant.

Next, we investigated the role of the N terminus, with a focus on the conserved N-terminal region, the so-called extended transmembrane Orai N-terminal (ETON) region (see Fig. 4 (*top*)), in the maintenance of CRAC channel characteristics of these constitutively active Orai mutants by generating analogue N-terminal deletions in both Orai1 and Orai3. We and others (27) have reported that among analogue Orai N-terminal deletion mutants (*i.e.* Orai1 ΔN_1–78_ and Orai3 ΔN_1–53_, only that of Orai3 remains functional upon STIM1-dependent activation (29). Correspondingly, when truncating the N terminus of the constitutive Orai mutants, the activity of Orai1 ΔN_1–78_ L185A/F250A and P245L mutants was abolished (65), whereas the analogue Orai3 N-terminal deletion mutants (Orai3 ΔN_1–53_ F160A and P254L) were still active (see Fig. 4, *k* and *p*). Although this has been interpreted (29, 59) as distinct N-terminal structural requirements in the STIM1-dependent activation of Orai1 and Orai3, the results in the accompanying paper (65) revealed that activation of non-functional Orai1 N-truncation mutants could be regained by switching loop2 with that of Orai3 (L2 aa 119–147). Because similar behavior was observed with the constitutively active Orai1 L185A/F250A and P245L mutants (see Fig. 4), the respective functional Orai1 Orai3-L2 chimeras were employed for comparison of the maintenance of CRAC channel hallmarks of Orai1 and Orai3 N-truncation mutants. Side effects from the swap of loop2 can be largely excluded, as full-length Orai1 Orai3-L2 and Orai1 Orai3-L2 P245L each co-expressed with STIM1 showed the expected current increases when switching from Ca^2+^ to DVF Na^+^ solution (Fig. S6, *a* and *b*).

Examination for the typical CRAC channel hallmarks of the constitutive Orai N-truncation mutants (Orai1 ΔN_1–78_ Orai3-L2 L185A F250A, Orai1 ΔN_1–78_ Orai3-L2 P245L, Orai3 ΔN_1–53_ F160A, and Orai3 ΔN_1–53_ P254L), both with and without STIM1, revealed that neither the increase in current size with DVF solution (Fig. 4, *a–d*, *f–i*, *k–n*, and *p–s*) nor the inactivation profile (Fig. 4, *e*, *j*, *o*, and *t*) was restored by the presence of STIM1, when a substantial portion of the Orai1 or Orai3 N terminus was missing. We confirmed coupling of Orai truncation mutants to STIM1 C-terminal fragments, as fluorescence intensity measurements revealed that co-localization was retained although somewhat reduced, as exemplarily shown of Orai3 ΔN_1–53_ F160A and the Orai1-activating STIM1 fragment (OASF) (Fig. S7).

In summary, all of the constitutively active Orai1 and Orai3 mutants with corresponding N-terminal deletions lacked the CRAC channel hallmarks, both in the absence and presence of STIM1. These results suggested that N-terminal residues located upstream of Lys^78^ in Orai1 and Arg^53^ in Orai3 were required for the STIM1-dependent maintenance of these CRAC channel hallmarks.

### The conserved ETON portion is required to maintain the CRAC channel hallmarks of constitutively active mutants in the presence of STIM1

**Figure 5.**
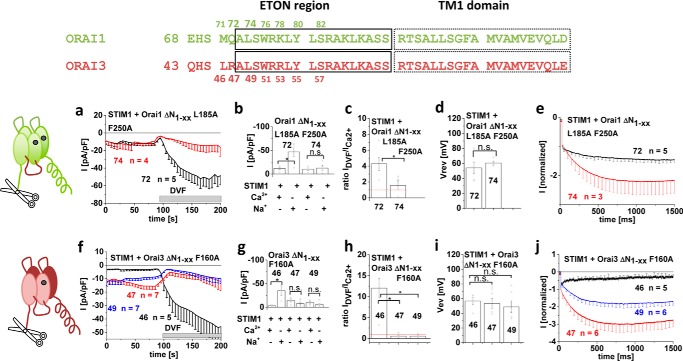
**Constitutively active Orai channels require the whole conserved ETON region to maintain CRAC channel hallmarks.** Shown are schemes illustrating the Orai channels with the respective mutations tested: Orai1 ΔN_1–72/74_ L185A/F250A and Orai3 ΔN_1–46/47/49_ F160A. *a* and *f*, respective time courses of whole-cell inward currents at −74 mV of constitutively active mutants: Orai1 ΔN_1–72/74_ L185A/F250A (*a*) and Orai3 ΔN_1–46/47/49_ F160A (*f*) in the absence compared with the presence of STIM1. At *t* = 0 s, inward currents in 10 mm extracellular Ca^2+^ solution activated upon passive store depletion via 10 mm EGTA are shown after reaching a steady-state level, and after 100 s, Na^+^-DVF solution was perfused. *b* and *g*, block diagram exhibiting current density at 50 and 150 s of tested mutants without and with STIM1 (*a* and *f*). *c* and *h*, block diagram exhibiting the ratio of *I*_DVF_ *versus I*_Ca_^2+^ of tested mutants without and with STIM1 (*a* and *f*). *d* and *i*, block diagram exhibiting the reversal potentials of mutants tested in *a* and *f* in the absence compared with the presence of STIM1. *e* and *j*, inactivation characteristics of constitutively active mutants shown in this figure in the absence compared with the presence of STIM1. *Error bars*, S.E.; *, *p* < 0.05; *n.s.*, not significant.

Next, we investigated in more detail to what extent the conserved ETON region (see Fig. 5 (*top*)) is required for maintaining the typical CRAC channel hallmarks in the presence of STIM1. We examined less pronounced N-truncations of constitutively active Orai1 channels with increasing deletions of ΔN_1–72_ and ΔN_1–74_. Orai1 ΔN_1–72_ L185A/F250A displayed authentic CRAC channel characteristics in the presence of STIM1 (Fig. 5*a*). Specifically, current densities increased following the DVF solution switch (Fig. 5, *a–e*), and current responses to voltage steps showed almost no potentiation, comparable with wildtype Orai channels (Fig. 5*c*). In contrast, the further deletion of two additional N-terminal residues (*i.e.* Orai1 ΔN_1–74_ L185A/F250A) led to a loss of these CRAC channel hallmarks in the presence of STIM1, resulting in substantially decreased current responses to DVF solution concomitant to a strong current potentiation (Fig. 5, *a–e*). Corresponding (Orai3 residues 47/49 correspond to Orai1 72/74) N-terminal deletions in Orai3 (Orai3 ΔN_1–47/49_ F160A) already resulted in a loss of the typical CRAC channel hallmarks for both constitutively active mutants in the presence of STIM1. However, N-terminal deletion, including residue 46, generated the mutant Orai3 ΔN_1–46_ F160A that retained all of the typical CRAC current hallmarks (Fig. 5, *f–j*). Thus, Orai3, in comparison with Orai1, needed the presence of one additional N-terminal residue upstream to the conserved N-terminal region to retain the typical CRAC channel characteristics (see alignment in Fig. 5 (*top*)).

In summary, the regulatory impact of STIM1 in conferring CRAC channel hallmarks to constitutively active TM3/TM4 Orai1 and Orai3 mutants required at least the presence of the whole, conserved N-terminal region (Orai1 aa 73–90; Orai3 aa 48–65) and, in the case of Orai3, one more upstream residue.

### Store-operated Orai channels exhibit identical structural N-terminal requirements to maintain authentic CRAC channel activity in the presence of STIM1

**Figure 6.**
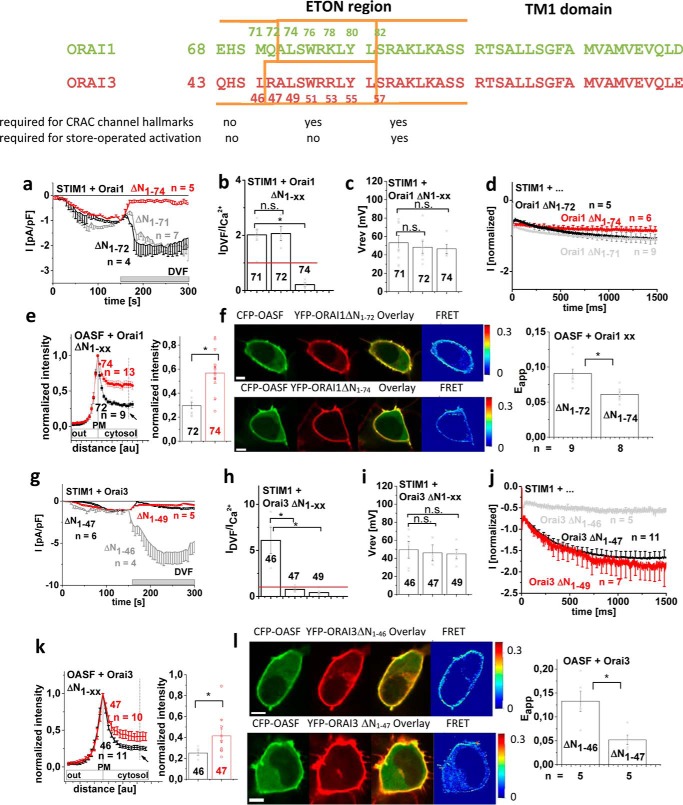
**STIM1-dependent store-operated Orai channel activation requires identical N-terminal portions as constitutively active Orai mutants to maintain CRAC channel hallmarks.** Shown is the sequence alignment of Orai1 (aa 68–110) and Orai3 (aa 43–115) in a comparison exhibiting which segments are required for maintenance of CRAC channel hallmarks. *a* and *g*, respective time courses of whole-cell inward currents at −74 mV of Orai1 ΔN_1–71/72/74_ (*a*) and Orai3 ΔN_1–46/47/49_ (*g*) in co-expression with STIM1. Inward currents activated upon passive store depletion via 10 mm EGTA are shown in 10 mm extracellular Ca^2+^ solution. After they reached a steady-state level at 150 s, Na^+^-DVF solution was perfused. *b* and *h*, block diagram exhibiting the ratio of *I*_DVF_ *versus I*_Ca_^2+^ of tested mutants without and with STIM1 (*a* and *f*). *c* and *i*, block diagram exhibiting the reversal potentials of mutants tested in *a* and *f* in the absence compared with the presence of STIM1. *d* and *j*, inactivation characteristics of constitutively active mutants shown in this figure in the absence compared with the presence of STIM1. *e* and *k* (*left*), intensity plots representing the localization of STIM1 OASF (aa 233–474) across the cell when co-expressed with Orai1 ΔN_1–72_ compared with Orai1 ΔN_1–74_ (*e*) and Orai3 ΔN_1–46_ compared with Orai3 ΔN_1–47_ (*k*). *Right*, block diagram exhibits corresponding mean and single values for normalized intensities at time point indicated by an *arrow* in the intensity plots on the *left. f* and *l* (*left*), image series depicting CFP-OASF and YFP-Orai1, -Orai1 ΔN_1–78_, or Orai1 ΔN_1–78_ Orai3-L2, *overlay* and *pixelwise* calculated *N*_FRET_ index for a representative cell. *Right*, the *yellow arrows* denote plasma membrane localization. A block diagram exhibits mean *N*_FRET_ determined from the averages of whole-cell areas for the respective number of cells expressing the depicted constructs. *Bars*, 5 μm. *Error bars*, S.E.; *, *p* < 0.05; *n.s.*, not significant.

In the following, we investigated whether store-operated Orai channels that were activated via STIM1 displayed authentic CRAC channel characteristics with the N-terminal deletions (see alignment in Fig. 6 (*top*)) previously identified with the constitutively active forms. Whereas STIM1-dependent currents of Orai1 ΔN_1–71_ or ΔN_1–72_ displayed the expected, yet less pronounced increase in current density upon switching from the Ca^2+^ to the DVF solution, Orai1 ΔN_1–74_ channels had already yielded a decrease (Fig. 6, *a–c*), consistent with the corresponding, constitutively active Orai1 mutants (see Fig. 5 (*a–c*)). Similarly, only Orai3 ΔN_1–46_ retained these typical CRAC hallmarks, whereas further N-terminal deletions to 47 and 49 resulted in reduced currents in DVF solution. Co-localization and FRET with STIM1-OASF was accordingly enhanced for Orai1 ΔN_1–72_ and Orai3 ΔN_1–46_ compared with Orai1 ΔN_1–74_ and Orai3 ΔN_1–47_, respectively (Fig. 6, *e*, *f*, *k*, and *l*). Interestingly, FCDI assessed by the response to a voltage step was not that much different between the Orai1 N-terminal deletion mutants, whereas the Orai3 N-terminal deletions with reduced currents in DVF solution exhibited the expected current potentiation (Fig. 6, *d* and *i*).

The finding that Orai1 required one amino acid less than Orai3 to maintain CRAC channel hallmarks suggested isoform-specific N-terminal requirements. In an attempt to understand this phenomenon, we utilized N-truncated Orai1 chimeras containing Orai3-L2 to mimic the Orai3 background. All generated N-truncated Orai1 Orai3-L2 chimeras with increasing deletions (Orai1 Orai3-L2 ΔN_1–71_, ΔN_1–72_, ΔN_1–74_, and ΔN_1–78_) lost their typical CRAC channel characteristics, which is in line with analogue Orai3 N-truncation mutants, except for Orai1 ΔN_1–71_ Orai3-L2 (Fig. S8). The latter was in contrast to the analogue Orai3 ΔN_1–46_, which exhibited enhanced *I*_DVF_ *versus I*_Ca_^2+^. Here, introducing the single point mutation Q72R into Orai1 Orai3-L2 ΔN_1–71_ to mimic the Orai3 N terminus portion restored typical CRAC channel characteristics (Fig. S8). These results further suggested communication between the Orai N-terminal region and Orai-L2 (see Ref. 65).

In aggregate, not only constitutive Orai mutants, but also store-operated Orai channels, required the presence of STIM1 and the analogue N-terminal portion to fully reconstitute CRAC channel hallmarks. In addition, the N-terminal portion necessary for these CRAC channel hallmarks was apparently longer than that required for retaining store-dependent activation (29, 59) (also see below).

### Mutations within the Orai1 N-terminal conserved region perturb CRAC channel characteristics

In addition to the N-terminal truncation mutants, we further tested the effect of two selected N-terminal point mutants, L74E/W76E (29) and K85E (60), for their effect on the typical CRAC channel hallmarks. Because both mutations interfered with STIM1-dependent Orai1 activation, their role in controlling CRAC channel hallmarks was tested in an Orai1 template with either Orai3-L2 and/or P245L in an attempt to restore Orai1 mutant channel activity.

**Figure 7.**
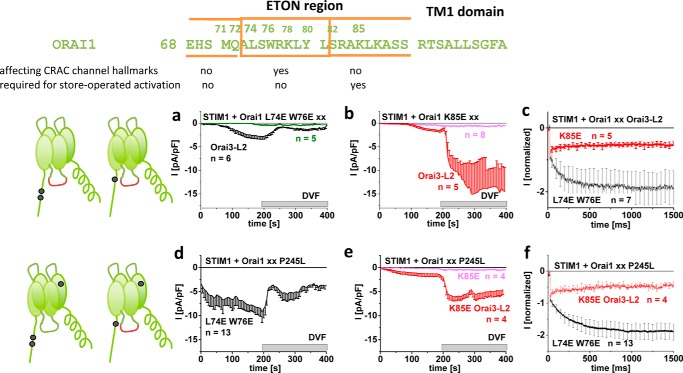
**N-terminal mutations within Orai1 perturb CRAC channel characteristics.** Shown is the sequence alignment of Orai1 (aa 68–110) exhibiting key residues involved in the maintenance of CRAC hallmarks or store-operated activation. Shown are schemes illustrating the Orai channels with the respective mutations tested: Orai1 L74E/W76E Orai3-L2, Orai1 L74E/W76E/P245L, Orai1 K85E Orai3-L2, and Orai1 K85E/P245L Orai3-L2. *a*, *b*, *d*, and *e*, time courses of whole-cell inward currents at −74 mV of Orai1 L74E/W76E Orai3-L2 in comparison with Orai1 L74E/W76E (*a*), Orai1 K85E Orai3-L2 compared with Orai1 K85E (*b*), Orai1 L74E/W76E/P245L (*d*), and Orai1 K85E/P245L compared with Orai1 K85E/P245L Orai3-L2 (*e*) in co-expression with STIM1. Inward currents activated upon passive store depletion via 10 mm EGTA are shown in 10 mm extracellular Ca^2+^ solution. After they reached a steady-state level, Na^+^-DVF solution was perfused. *c* and *f*, inactivation characteristic of the mutants shown in *a*, *b*, *d*, and *e. Error bars*, S.E.

STIM1-mediated activation of Orai1 L74E/W76E was restored upon the swap of Orai1-L2 for Orai3-L2 (Orai1 L74E/W76E Orai3-L2). Moreover, Orai1 L74E/W76E became constitutively active upon introduction of P245L (Orai1 L74E/W76E/P245L). However, both mutants with restored activity did not display any CRAC channel hallmarks (Fig. 7, *a–c*) in the presence of STIM1, in contrast to Orai1 Orai3-L2 (Fig. S6*a*) and Orai1 P245L (Fig. 1), suggesting Leu^74^/Trp^76^ as critical N-terminal determinants in the maintenance of authentic CRAC channel activity.

As progressive Orai1 N-truncations impaired initially the CRAC channel hallmarks followed by STIM1-dependent, store-operated activation, we further examined whether these features occurred independent of each other. Hence, we employed the K85E N-terminal point mutation, which interfered with STIM1-dependent activation (60), but left the rest of the N terminus intact. By swapping Orai1-L2 for Orai3-L2 in Orai1 K85E, we succeeded in slightly restoring STIM1-induced current activation (Fig. 7*b*), providing the opportunity to test for the CRAC channel hallmarks. Orai1 K85E Orai3-L2 displayed authentic CRAC channel characteristics comparable with Orai1 Orai3-L2 or wildtype Orai1 (Fig. S6*a*), in contrast to Orai1 L74E/W76E Orai3-L2. Furthermore, whereas Orai1 K85E/P245L remained inactive, an additional swap of Orai3-L2 (Orai1 K85E/P245L Orai3-L2) restored channel activation upon STIM1 co-expression, not in a constitutive but in a store-operated manner. Here, Orai1 K85E/P245L Orai3-L2 also exhibited fully preserved authentic CRAC channel hallmarks (Fig. 7, *d–e*), comparable with Orai1 P245L or Orai1 P245L Orai3-L2 in the presence of STIM1. Hence, whereas the extent of STIM1-dependent activity of Orai1 K85E can only be marginally restored by P245L or Orai3-loop2, the CRAC channel hallmarks remained unaffected by K85E localized to the second half of the conserved Orai1 N-terminal region (see alignment in Fig. 6 (*top*)).

In summary, we determined that distinct Orai1 N-terminal domains control typical CRAC channel characteristics and store-operated activation. Whereas the two N-terminal residues Leu^74^ and Trp^76^ were required to maintain the CRAC channel hallmarks, they were insignificant for store-operated STIM1-dependent activation. Lys^85^, however, controlled store-operated activation of Orai1 channels but did not affect the CRAC channel hallmarks. Thus, maintenance of the typical CRAC channel hallmarks required the first half of the conserved N-terminal region, whereas the second half was imperative for store-operated CRAC channel activation.

## Discussion

The constitutively active Orai TM3/TM4 mutants did not recapitulate authentic CRAC channel activity, which included reduced Ca^2+^ selectivity, distinctly different current responses to DVF-Na^+^ solution, and a lack of FCDI. Restoration of these CRAC channel hallmarks was obtained by coupling to STIM1 but failed when the conserved portion of Orai N terminus (ETON region) was either truncated or mutated. Generally, maintaining authentic CRAC channel activity required the presence of both STIM1 and the ETON region, not only with the constitutively active Orai mutants but also with wildtype Orai channels.

Although the constitutive TM3/TM4 Orai mutants presented here exhibited modestly reduced Ca^2+^ selectivity, they were much more selective than the constitutively active Orai1 V102A (34) form. CRAC channel-like Ca^2+^ selectivity could be restored by the presence of STIM1, as is common for these constitutively active Orai1 mutants. This high Ca^2+^ selectivity, also found in wildtype Orai1 channels, is ensured as long as a sufficient amount of STIM1 is bound (34). Additionally, STIM1 is indispensable for FCDI of Orai channels. In the absence of STIM1, the constitutively active TM3/TM4 Orai mutants completely lacked FCDI. Moreover, currents exhibited strong potentiation in response to a voltage step, reaching a plateau after 1500 ms, as consistently observed with other constitutively active Orai1 channels (34, 40). This potentiation apparently involved a Ca^2+^-dependent process, as it was observed neither with Ba^2+^ nor with Na^+^ as charge carriers, as exemplarily shown with Orai3 F160A (Fig. S9, *a* and *b*). Hence, STIM1 reverses this potentiation into FCDI, thereby limiting physiological Ca^2+^ entry into the cell, re-establishing this hallmark of authentic CRAC channel activity.

The current increase when switching from the Ca^2+^ to the DVF Na^+^ solution represented another hallmark of authentic CRAC channel activity, which was drastically altered with the constitutively active TM3/TM4 mutant Orai channels in the absence of STIM1. Until now, several other TM1/TM2/TM3 Orai1 mutants like Orai1 G98D/S (33, 61), Orai1 F99S/M/Y (61), Orai1 V102C/A (34), Orai1 V107M (36), Orai1 L138F (37), Orai1 W176C (38), and Orai1 T184M (36) have been reported to induce constitutive activity in the absence of STIM1. Among those that have been examined for permeation in DVF Na^+^ solution, current increases are obtained with the Orai1 G98S, Orai1 F99Y, Orai1 V102A, and Orai1 W176C mutants (34, 61) upon the switch from a Ca^2+^- to Na^+^-containing solution. This is in contrast to what we observed with the constitutively active Orai TM3/TM4 mutants studied here. Hence, Na^+^ permeation of the former Orai1 mutants is not substantially hampered, possibly due to a better stabilization of the Orai1 channel open state, whereas the latter TM3/TM4 mutants studied here required both the presence of STIM1 and the conserved Orai N-terminal portion. The switch from a Ca^2+^-containing to a DVF Cs^+^-containing solution revealed current reductions for our Orai TM3/TM4 mutants, as exemplarily shown for Orai3 F160A in line with the lack of Cs^+^ permeation of typical CRAC currents (Fig. S9, *c* and *d*). In contrast, Orai1 V102A is permeable for Cs^+^ (34), which suggests a larger pore diameter for Orai1 V102A compared with Orai1 TM3/TM4 constitutive point mutants. The Orai1 V102A mutant consistently retains constitutive activity even with extensive N-terminal truncations (27, 29, 34, 40).

Only the fully conserved ETON region in Orai1 (aa 73–90) and in Orai3 (aa 47–65) could retain authentic CRAC channel activity by STIM1, as was the case in constitutively active as well as store-operated Orai channels. Even N-terminal truncations within this region that did not affect the principal activity were typically accompanied by the loss of STIM1-dependent CRAC channel hallmarks such as current increases in DVF Na^+^-containing solution and FCDI. Within this conserved N-terminal region in Orai1, Leu^74^ and Trp^76^, among other residues, played an important role, as their mutation similarly led to a loss of these CRAC channel hallmarks. However, the general activity of Orai channels, induced either via STIM1 or TM3/TM4 point mutations in a store-dependent or constitutive manner, respectively, only required the presence of the latter 10 residues (Orai1 aa 80–90; Orai3 aa 55–65) (29, 59) of the conserved N-terminal region. In line with this, introduction of the K85E mutation in full-length Orai1 that substantially impaired the STIM1-dependent, general Orai1 activity did not affect the typical CRAC channel hallmarks. Hence, distinct domains within the conserved Orai N-terminal portion convey different functions to the overall CRAC channel activity. Whereas the latter stretch (Orai1 aa 80–90; Orai3 aa 55–65) contributes to the general activity, the former part (Orai1 aa 73–79; Orai3 aa 47–54) is key for the CRAC channel hallmarks in fine-tuning Ca^2+^ entry. It thus far remains unclear whether these regulatory events occur via STIM1 interaction with the conserved Orai N terminus region (20, 29, 39, 62) or in an allosteric manner, with the Orai1 C terminus triggering signal transmission through TM domain reorientations (31) or through involvement of other parts of the channel protein (61). Within this context, a permissive communication between the Orai1 N terminus and loop2 (see Ref. 65) is required for retaining Orai1 channel activity, particularly with Orai1 N-terminal truncations and mutations within the aa 73–79 domain.

In summary, STIM1 communication with the conserved portion of the Orai N terminus (ETON region) is essential for maintaining the typical CRAC channel hallmarks. Whereas point mutations in TM3/TM4 generate constitutively active Orai channels, authentic CRAC channel activity requires the presence of STIM1 and the ETON region, as is similar with wildtype Orai channels. STIM1 binding to the Orai channel mechanistically alters the overall conformation and orientation of TM helices, including the ETON region, to manifest authentic CRAC channel gating and open pore structure (40, 61, 62). For more detained resolution of the STIM-Orai complex, molecular dynamics simulations or single-particle cryo-EM are required.

## Experimental procedures

### Molecular biology

For N-terminal fluorescence labeling of human Orai1 (Orai1; accession number NM_032790, provided by the laboratory of A. Rao) as well as Orai3 (Orai3; accession number NM_152288, provided by the laboratory of L. Birnbaumer), the constructs were cloned into the pEYFP-C1 (Clontech) expression vector via KpnI and XbaI (Orai1) and BamHI and XbaI (Orai3) restriction sites, respectively. Orai1 N-terminal deletion mutants (Orai1 ΔN_1–71_, ΔN_1–72_, ΔN_1–74_, ΔN_1–76_, and ΔN_1–78_) were amplified via PCR, including an N-terminal KpnI and a C-terminal XbaI restriction site; Orai3 N-terminal deletion mutants (Orai3 ΔN_1–46_, ΔN_1–47,_ ΔN_1–49_, and ΔN_1–53_) were amplified via PCR, including an N-terminal BamHI and a C-terminal XbaI restriction site for cloning into the pEYFP-C1 vector. Chimeric constructs (Orai1-Orai3-loop2 and Orai1 ΔN_1–78_-Orai3-loop2) were cloned via SOEing (Splicing by Overlap Extension) into the pEYFP-C1 (Clontech) expression vector for N-terminal fluorescence labeling. Site-directed mutagenesis (L185A/F250A, P245L, K85E/P245L, L74E/W76E/P245L, K85E, L74E/W76E, and P245L/L273S/D) was performed using the QuikChange^TM^ XL site-directed mutagenesis kit (Stratagene) with the corresponding Orai1 and/or Orai1-Orai3 chimeric constructs serving as a template. The same procedure was used for Orai3 (F160A and P254L). For C-terminal fluorescence labeling of human Orai1 (Orai1; accession number NM_032790, provided by the laboratory of A. Rao), the construct was cloned into the pEYFP-N1 (Clontech) expression vector via XhoI and BamHI restriction sites.

Human STIM1 (STIM1; accession number NM_003156) N-terminally ECFP-tagged was kindly provided by the laboratory of T. Meyer (Stanford University). pECFP-C1 STIM1 C terminus (aa 233–685 WT and L251S) was used as a template for generation of pECFP-OASF (WT and L251S) by introducing a stop codon at position 475 (aa 233–474) using the QuikChange XL site-directed mutagenesis kit (Stratagene). STIM1 fragment 344–449 (CRAC activation domain) was amplified via PCR, including an N-terminal KpnI and a C-terminal XbaI restriction site for cloning into the pECFP-C1 vector.

All clones were confirmed by sequence analysis.

### Cell culture and transfection

Transient transfection of HEK 293 cells was performed (63) using either the TransFectin lipid reagent (Bio-Rad) or the TransPass transfection reagent (New England Biolabs). The CRISP/Cas9 STIM1 knockout HEK cells were kindly provided by M. Trebak (Penn State).

### Electrophysiology

Electrophysiological recordings that assessed the characteristics of 2–3 constructs were carried out in paired comparison on the same day. Expression patterns and levels of the various constructs were carefully monitored by confocal fluorescence microscopy and were not significantly changed by the introduced mutations. Electrophysiological experiments were performed at 20–24 °C, using the patch-clamp technique in the whole-cell recording configuration. For STIM1/Orai as well as STIM1 C terminus/Orai current measurements, voltage ramps were usually applied every 5 s from a holding potential of 0 mV, covering a range of −90 to +90 mV over 1 s. Voltage step protocols were applied from a holding potential of 0 to −70 mV for 1.5 s to determine FCDI. The internal pipette solution for passive store depletion contained 3.5 mm MgCl_2_, 145 mm cesium methane sulfonate, 8 mm NaCl, 10 mm HEPES, 20 mm EGTA, pH 7.2. Extracellular solution consisted of 145 mm NaCl, 5 mm CsCl, 1 mm MgCl_2_, 10 mm HEPES, 10 mm glucose, 10 mm CaCl_2_, pH 7.4. Na^+^-DVF solution contained 150 mm NaCl, 10 mm HEPES, 10 mm glucose, and 10 mm EDTA, pH 7.4. Applied voltages were not corrected for the liquid junction potential, which was determined as +12 mV. All currents were leak-corrected by subtraction of the leak current that remained following 10 μm La^3+^ application.

### Confocal fluorescence microscopy

Confocal microscopy for co-localization experiments was performed in a manner similar to that described previously (64). In brief, a QLC100 real-time confocal system (VisiTech International, Sunderland, UK) was used for recording fluorescence images connected to two Photometrics CoolSNAPHQ monochrome cameras (Roper Scientific) and a dual port adapter (dichroic: 505lp; cyan emission filter: 485/30; yellow emission filter: 535/50; Chroma Technology Corp.). This system was attached to an Axiovert 200M microscope (Zeiss, Jena, Germany) in conjunction with an argon ion multiwavelength (457, 488, and 514 nm) laser (Spectra Physics). The wavelengths were selected by an Acousto Optical tunable filter (VisiTech International). MetaMorph version 5.0 software (Universal Imaging Corp.) was used to acquire images and to control the confocal system. Illumination times for CFP and YFP images that were consecutively recorded with a minimum delay were about 900 ms.

### Statistics

Results are presented as means ± S.E. calculated for the indicated number of experiments. Student's two-tailed *t* test was used for statistical comparison considering differences statistically significant at *p* < 0.05.

## Author contributions

I. D. and C. R. conceived and coordinated the study and wrote the paper. I. D., C. B., A. K., and M. S. performed and analyzed electrophysiological experiments. M. M. carried out fluorescence microscopy experiments. C. B., M. F., and I. F. contributed to molecular biology. All authors reviewed the results and approved the final version of the manuscript.

## Supplementary Material

Supporting Information

## Footnotes

3 The abbreviations used are:

CRAC: Ca^2+^ release-activated Ca^2+^
STIM1: stromal interaction molecule 1
ER: endoplasmic reticulum
PM: plasma membrane
TM: transmembrane domain(s)
DVF: divalent-free
FCDI: fast Ca^2+^-dependent inactivation
ETON: extended transmembrane Orai N-terminal
aa: amino acids
OASF: Orai1-activating STIM1 fragment
CFP: cyan fluorescent protein
ECFP: enhanced CFP
YFP: yellow fluorescent protein
L2: loop2.
