# Supplementary material for: Authentic CRAC channel activity requires STIM1 and the conserved portion of the Orai N terminus
Source: J Biol Chem. 2017 Dec 13;293(4):1259–70. doi: 10.1074/jbc.M117.812206 (PMC5787803; doi:10.1074/jbc.M117.812206)
Supplement: Supporting Information [file supp_293_4_1259__index.html]

Authentic CRAC channel activity requires STIM1 and the conserved portion of the Orai N-terminus — Authentic CRAC channel activity requires STIM1 and the conserved portion of the Orai N terminus — CRAC channel activity requires STIM1 and Orai N terminus — Supporting Information 

# Authentic CRAC channel activity requires STIM1 and the conserved portion of the Orai N terminus

## Supporting Information

- Suppl Figs. 1-9 (.pdf, 767 KB) - Suppl Figs. 1-9 including legends
