# Supplementary material for: Authentic CRAC channel activity requires STIM1 and the conserved portion of the Orai N terminus
Source: J Biol Chem. 2017 Dec 13;293(4):1259–70. doi: 10.1074/jbc.M117.812206 (PMC5787803; doi:10.1074/jbc.M117.812206)

Supplementary Figures

Supplementary Figure 1: Comparison of the CRAC channel characteristics of Orai1 and Orai3. a) g) Respective time courses of whole-cell inward currents at -74 mV of Orai1 (a), Orai3 (g) in co-expression with STIM1. Inward currents activated upon passive store-depletion via 10mM EGTA are shown in 10 mM extracellular Ca^{2+} solution. After they reached a steady state level at 150 s Na^{+}-DVF was perfused. **b) h)** Block diagram exhibiting current density at 50s and 150s of tested mutants without and with STIM1 (a, g). **e) k)** Block diagram exhibiting the ratio of I_{DVF} versus I_{Ca2+} of tested mutants without and with STIM1 (a, g). **d) j)** I/V relationships of Orai1 and Orai3 currents in 10mM Ca^{2+} containing and Na^{+}-DVF solution. **f) l)** Inactivation characteristics of STIM1-mediated Orai1 and Orai3 currents.

Supplementary Figure 2: Current-voltage relationships of constitutive Orai channels in Ca^{2+} and DVF solution, without and with STIM1 co-expressed. a) - h) I/V relationships of Orai1 L185A F250A, Orai1 P245L, Orai3 F160A and Orai3 P254L currents without and with STIM1 co-expressed in 10mM Ca^{2+} containing compared to Na^{+}-DVF solution.

Supplementary Figure 3: C-terminally labelled Orai1 P245L exhibits analogue results like the N-terminally labelled one. a) Respective time courses of whole-cell inward currents at -74 mV of the C-terminally labelled Orai1 P245L (a). At t = 0 s inward currents in 10 mM extracellular Ca^{2+} solution activated upon passive store-depletion via 10mM EGTA are shown after reaching a steady state level and after 100s Na^{+}-DVF was perfused. **b)** On the right a block diagram exhibits the current density at 50s and 150s of tested mutants, respectively. **c)** Block diagram exhibiting the ratio of I_{DVF} versus I_{Ca2+} of tested mutants without and with STIM1. **d)** Inactivation profile of C-terminally labelled Orai1 P245L currents in the absence compared to the presence of STIM1.

Supplementary Figure 4: Impaired coupling of STIM1 to a constitutive Orai1 mutants results in abolished CRAC hallmarks. a) Time courses of whole-cell inward currents at -74 mV of Orai1 P245L in the absence and presence of STIM1 in CRISP/Cas9 STIM1 knock-out HEK 293 cells. **b)** Block diagram exhibiting current density at 50s and 150s of tested mutants (a) without and with STIM1. **c)** Block diagram exhibiting the ratio of I_{DVF} versus I_{Ca2+} of tested mutants without and with STIM1 (a). **d)** Time courses of whole-cell inward currents at -74 mV of Orai1 P245L L273D in the absence and presence of STIM1. **e)** Block diagram exhibiting current density at 50s and 150s of tested mutants (d) without and with STIM1. **f)** Block diagram exhibiting the ratio of I_{DVF} versus I_{Ca2+} of tested mutants without and with STIM1 (d). **g) h)** (left) Intensity plots representing the localization of STIM1 OASF (233-474) (g) or STIM1 OASF L251S (h) across the cell when co-expressed with Orai1 P245L compared to Orai1 P245L L273D. (right) Block diagram exhibits corresponding mean and single values for normalized intensities at time point indicated by an arrow in the intensity plots on the left.

Supplementary Figure 5: Orai3 F160A currents monitored upon ramps for t = 200ms. a) Respective time courses of whole-cell inward currents at -74 mV of the C-terminally labelled constitutively active mutants Orai3 F160A. At t = 0 s inward currents in 10 mM extracellular Ca^{2+} solution activated upon passive store-depletion via 10mM EGTA are shown after reaching a steady state level and after 100s Na^{+}-DVF was perfused. **b)** Block diagram exhibiting current density at 50s and 150s of tested mutants (a) without and with STIM1. **c)** Block diagram exhibiting the ratio of I_{DVF} versus I_{Ca2+} of tested mutants without and with STIM1 (a). **d) e)** I/V relationships of Orai3 F160A currents without and with STIM1

co-expressed in 10mM Ca^{2+} containing compared to Na^{+}-DVF solution. **f)** Block diagram exhibiting the reversal potentials of the respective mutants in a).

Supplementary Figure 6: Full-length Orai1-Orai3-L2 chimeras exhibit when co-expressed with STIM1 maintained CRAC hallmarks. a) move from figure before to this figure Respective time courses of normalized whole-cell inward currents at -74 mV of Orai1, Orai1 ΔN_{1-72}, Orai1-Orai3-L2 in co-expression with STIM1. Inward currents activated upon passive store-depletion via 10mM EGTA are shown in 10 mM extracellular Ca^{2+} solution. After they reached a steady state level at ~150 s Na^{+}-DVF was perfused. **b)** Time courses of normalized whole-cell inward currents at -74 mV of Orai1-Orai3-L2 P245L in co-expression with STIM1 activated upon passive store-depletion. Upon maximal activation at ~100s Na^{+}-DVF was perfused.

Supplementary Figure 7: STIM1 OASF and STIM1 OASF L251S are co-localized with Orai3 ΔN_{1-53} F160A. a) b) (left) Intensity plots representing the localization of STIM1 233-474 (a) and STIM1 233-474 L251S (b) across the cell when co-expressed with Orai3, Orai3 ΔN_{1-53} and Orai3 F160A and Orai3 ΔN_{1-53} F160A. (right) Block diagram exhibits corresponding mean and single values for normalized intensities at time point indicated by an arrow in the intensity plots on the left.

Supplementary Figure 8: Only Orai1 ΔN_{1-71} Q72R Orai3-L2 exhibits maintained CRAC hallmarks in the presence of STIM1. a) Respective time courses of whole-cell inward currents at -74 mV of Orai1 $\Delta N_{1-71/72/74/78}$ Orai3-L2 and Orai1 ΔN_{1-71} Q72R Orai3-L2 in co-expression with STIM1. Inward currents activated upon passive store-depletion via 10mM EGTA are shown in 10 mM extracellular Ca^{2+} solution. After they reached a steady state level at 150 s Na^{+}-DVF was perfused. **b)** Inactivation profile of mutants in (a) in the presence of STIM1 in comparison.

Supplementary Figure 9: Inactivation profiles of Orai 3 F160A with **a)** Ba2+ or **b)** Na+ as permeant ion in the absence as well as presence of STIM1. **c)** Respective time courses of whole-cell inward currents at -74 mV of Orai3 F160A in the absence and presence of STIM1. Inward currents activated upon passive store-depletion via 10mM EGTA are shown in 10 mM extracellular Ca^{2+} solution. After they reached a steady state level at 150 s Cs^{+}-DVF was perfused. **d)** Block diagram exhibiting current density at 50s and 150s of tested mutants without and with STIM1 (c).

Supplementary Figure 1

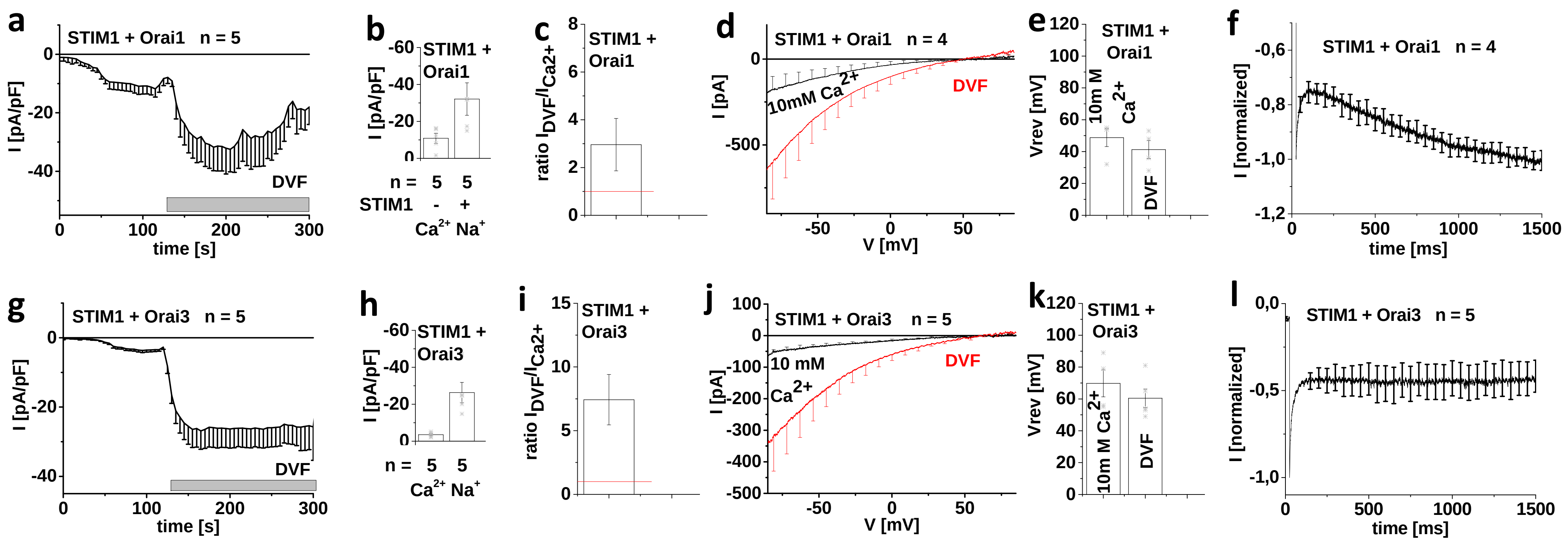

Supplementary Figure 2

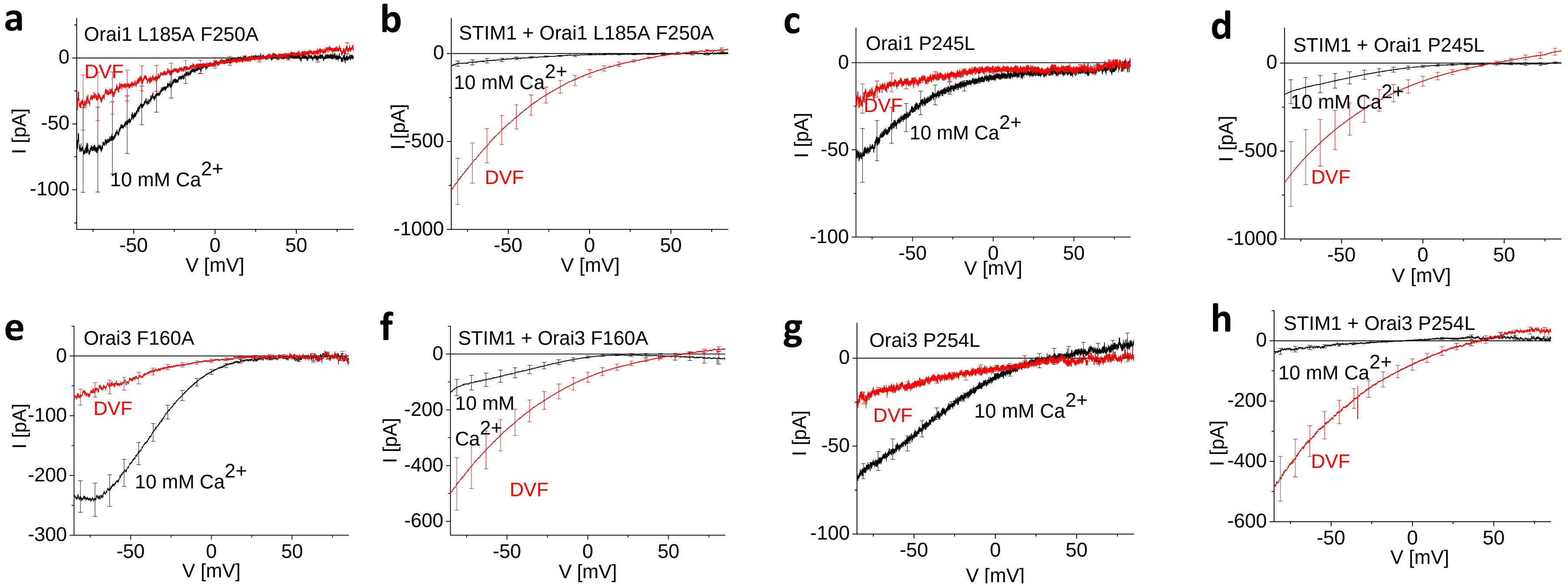

Supplementary Figure 3

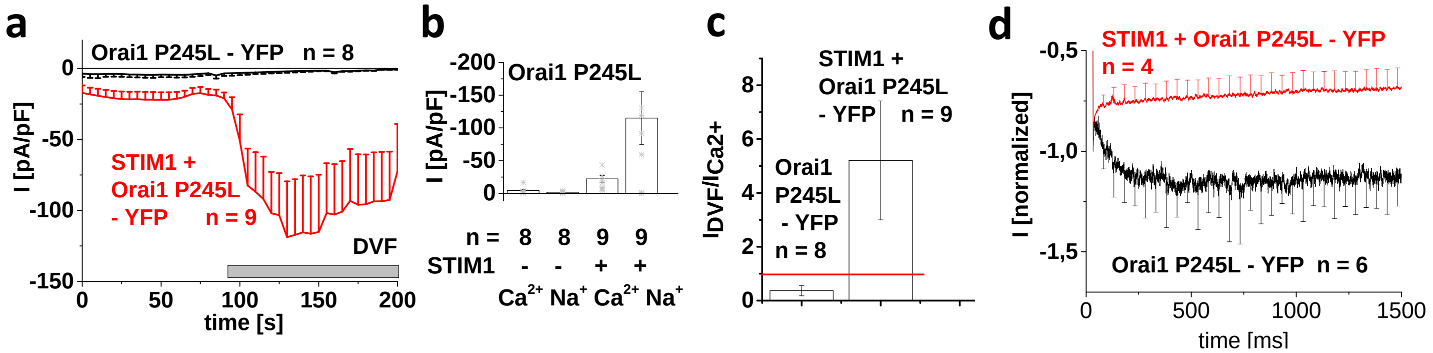

Supplementary Figure 4

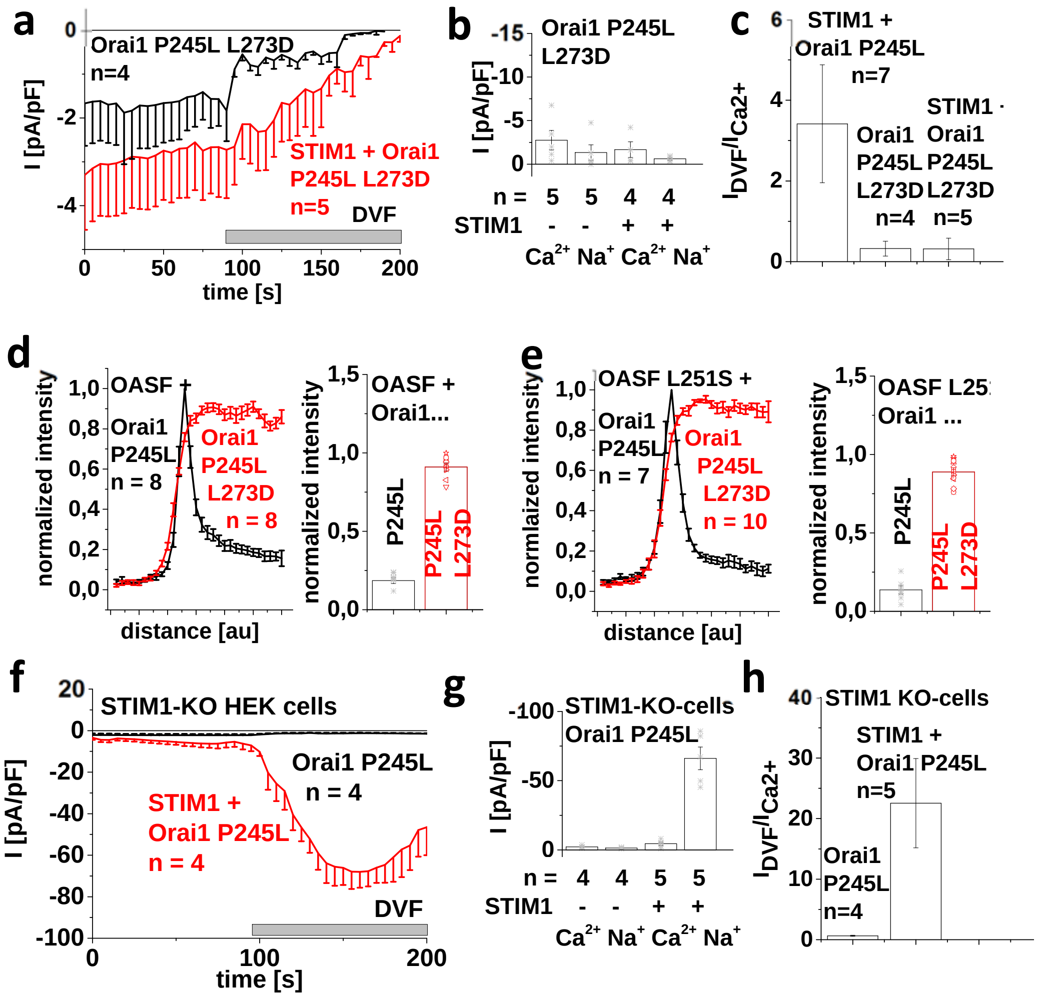

Supplementary Figure 5

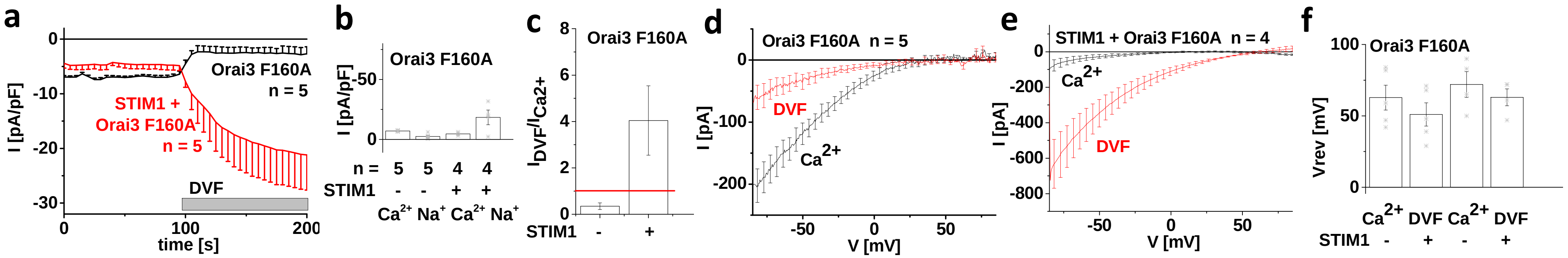

Supplementary Figure 6

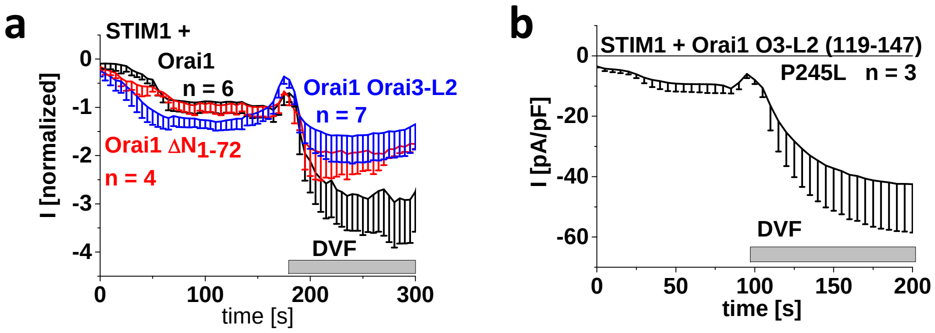

Supplementary Figure 7

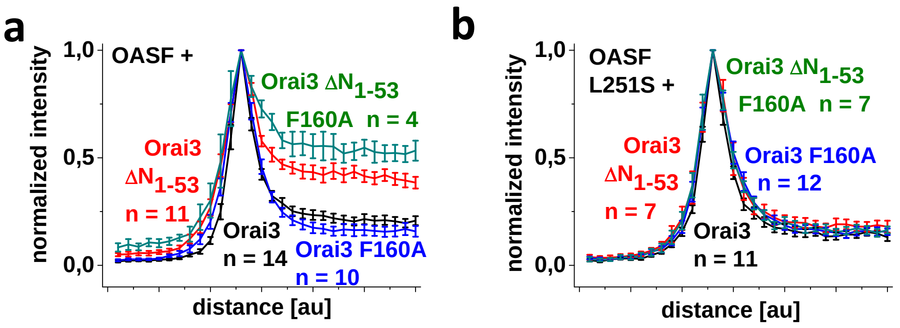

Supplementary Figure 8

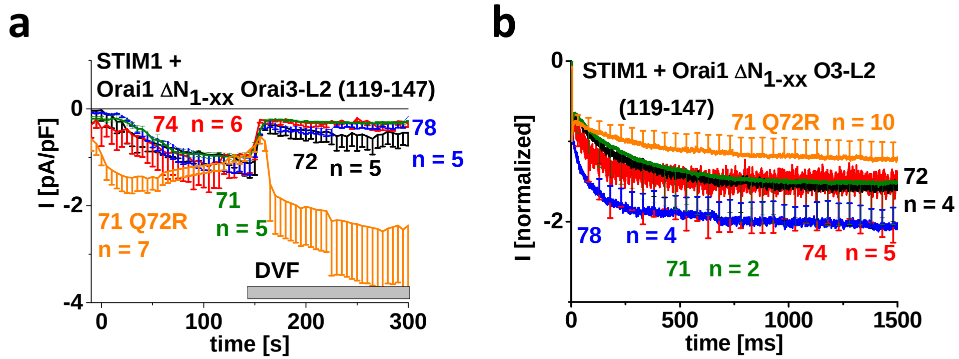

Supplementary Figure 9

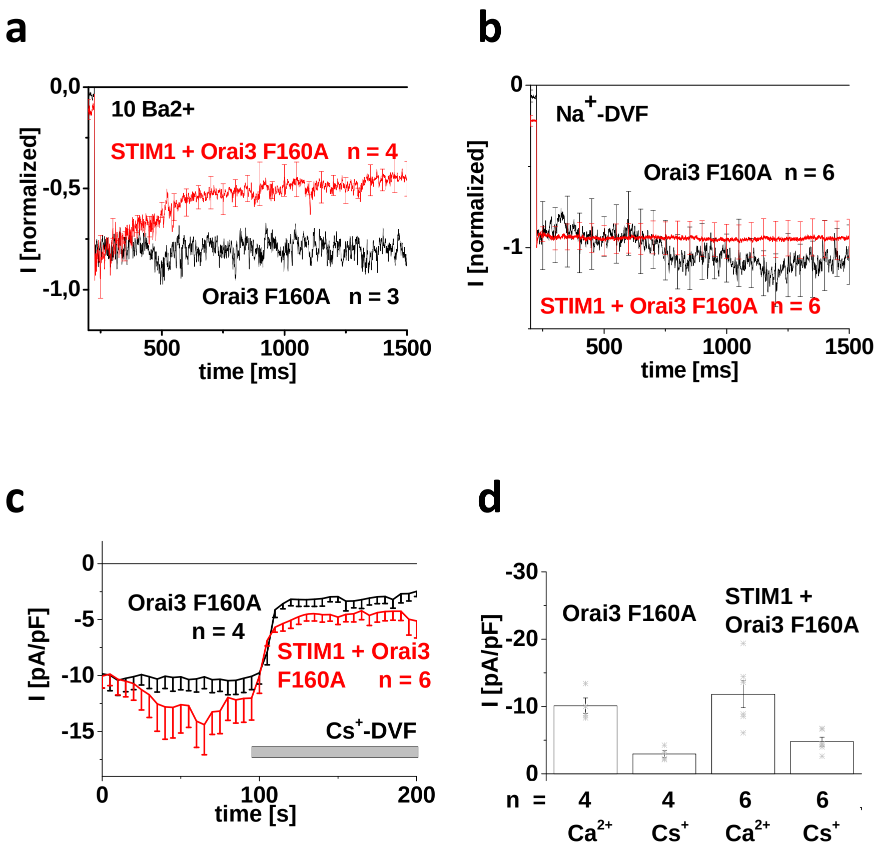

Supplement: Supporting Information [file 10.1074_M117.812206_jbc.M117.812206-1.pdf]
